# Investigation on the effects of the atmospheric pressure plasma on wound healing in diabetic rats

**DOI:** 10.1038/srep19144

**Published:** 2016-02-23

**Authors:** Sara Fathollah, Shahriar Mirpour, Parvin Mansouri, Ahmad Reza Dehpour, Mahmood Ghoranneviss, Nastaran Rahimi, Zahra Safaie Naraghi, Reza Chalangari, Katalin Martits Chalangari

**Affiliations:** 1 Plasma Physics Research Center, Science and Research branch of Islamic Azad University, Tehran, Iran; 2 Laser and Plasma institute, Shahid Beheshti University, Tehran, Iran; 3 Skin and Stem cell Research Center, Tehran University of Medical Sciences, Tehran, Iran; 4 Experimental Medicine Research Center, Tehran University of Medical Science, Tehran, Iran; 5 Department of Pharmacology, School of Medicine, Tehran University of Medical Sciences, Tehran, Iran; 6 Department of Pathology, Razi Skin Hospital, Tehran University of Medical Sciences, Tehran, Iran; 7 Kassir Dermatology, 8335 Walnut Hill Ln, #140, Dallas, TX 75231, USA

## Abstract

It is estimated that 15 percent of individuals with diabetes mellitus suffer from diabetic ulcers worldwide. The aim of this study is to present a non-thermal atmospheric plasma treatment as a novel therapy for diabetic wounds. The plasma consists of ionized helium gas that is produced by a high-voltage (8 kV) and high-frequency (6 kHz) power supply. Diabetes was induced in rats via an intravascular injection of streptozotocin. The plasma was then introduced to artificial xerograph wounds in the rats for 10 minutes. Immunohistochemistry assays was performed to determine the level of transforming growth factor (TGF-β1) cytokine. The results showed a low healing rate in the diabetic wounds compared with the wound-healing rate in non-diabetic animals (P < 0.05). Moreover, the results noted that plasma enhanced the wound-healing rate in the non-diabetic rats (P < 0.05), and significant wound contraction occurred after the plasma treatment compared with untreated diabetic wounds (P < 0.05). Histological analyses revealed the formation of an epidermis layer, neovascularization and cell proliferation. The plasma treatment also resulted in the release of TGF-β1 cytokine from cells in the tissue medium. The findings of this study demonstrate the effect of plasma treatment for wound healing in diabetic rats.

Wound healing is a common concern in diabetic patients. Patients with diabetes have wound healing complications associated with many factors, including neuropathy, vascular disease, and foot deformities^1^. The conventional therapeutic strategies are limited to wound dressings, cell therapy, and oxygen therapy. These methods are not completely successful in curing diabetic wounds because of the slow wound healing process, the high cost of therapies, and the lack of improvement in the disturbed healing process caused by diabetes^2^. Non-thermal atmospheric pressure plasma, which has been published as a possible new treatment in chronic wound therapy, may hopefully solve this issue.

Plasmas are partially-ionized gases and are described as the fourth state of matter. The word “plasma” was coined by Irving Langmuir, for the ionized gas which is comprised of suspended electrons, ions, and other excited and charged particles^3^,^4^. Medical applications of non-thermal plasma are classified into direct plasma and indirect plasma therapy; the former is used directly on the human body, while the later is generated remotely and then transported to the surface to be treated^5^. Plasma application creates a new field (at the precinct) between plasma science and biomedicine. In recent years, atmospheric pressure plasma has demonstrated many potential applications in skin wound healing^6^, blood coagulation^7^,^8^, bacteria disinfection^9^,^10^ and cancer therapy^11^,^12^. The possibility of potentiation of wound healing by atmospheric plasma has been the focus of attention in many recent studies^13^,^14^,^15^. Plasma can produce reactive species such as Nitric oxide (NO), Hydroxyl (OH) and Atomic oxygen (O) which is an important reaction by living microorganisms^16^. This special tissue-reactive species interaction may lead to the acceleration of tissue repair processes without adverse effects on normal tissue thereby differentiating this technique from other conventional methods^17^. There are many publications on cold plasma therapy and its significance; however, they suffer from the lack of data on the effectiveness of this technique on metabolic ulcers.

The aim of this study is to investigate the effect of plasma treatment on diabetic wounds in rat models as well as the possible mechanism of the plasma-wound interaction.

## Results

### Blood glucose level of animals

The blood glucose levels of the animals during the experiment were reported in Table 1. The obtained data show that the STZ chemicals induced diabetes in the diabetic rat groups in all of the experiment days (P > 0.05). Surprisingly, the results indicate that plasma treatment reduced the blood glucose level in the diabetic group compared to the non-treated control (P < 0.05).

### Effect of diabetes on healing of wound contraction on the rats

**Figure 1:**
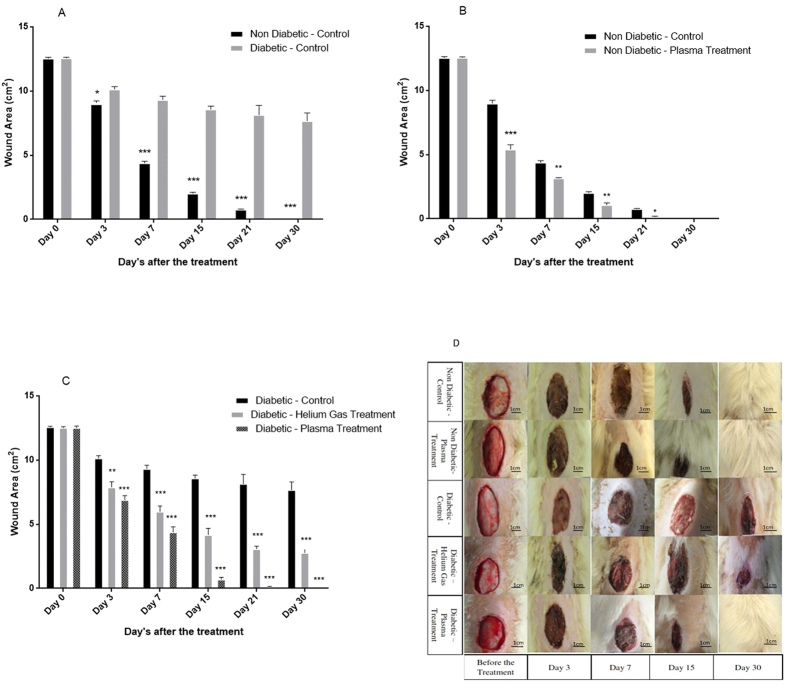
(**A**) Wound area measurement in non-diabetic and diabetic rats during days 3, 7, 15, 21 and 30 after the plasma treatment. Wound area expressed in cm^2^ mean ± S.D. (*p < 0.05, ***p < 0.001-compared with the control in same day). (**B**) Change in the wound closure area at days 3, 7, 15, 21 and 30 after the plasma treatment in non-diabetic models. Wound area expressed in cm^λ^ mean ± S.D. (***p < 0.001 (compared with control in same day), **p < 0.01, *p < 0.05). (**C**) Change in the wound closure area at days 3, 7, 15, 21 and 30 after the plasma treatment in diabetic models. Wound area expressed in cm^2^ and mean ± S.D. (**p < 0.01, ***p < 0.001-compared with control in each day). (**D**) Wound observation for days 3,7,15 and 30 after the treatment.

Figure 1(A) shows the wound contraction area that has healed in diabetic and non-diabetic models without any treatment. The wound area was reduced significantly in the non-diabetic groups 3 days after the wound creation (t(10) = 2.22, H.R: 45%, P < 0.05). This difference is considerable 7 and 15 days after the wound creation (t(10) = 4.58, H.R = 57%, P < 0.001 −t(10) = 4.58, H.R = 97%, P < 0.001 respectively).

### Effect of non-thermal plasma on wounds on non-diabetic rats

Figure 1(B) shows the effect of non-thermal atmospheric pressure plasma on ulcers in non-diabetic and diabetic animals. The results show that plasma treatment accelerated the wound healing in non-diabetic rats (P < 0.001). The wound area was reduced significantly 3 and 7 days after the plasma treatment (t(10) = 4.58, H.R: 50%, P < 0.001 −t(10) = 3.16, H.R: 75%, P < 0.01 respectively).

### Effect of non-thermal plasma on wounds on diabetic rats

The wound area in plasma and helium gas treated animals were calculated and reported in Fig. 1(C). This figure shows that 3 days after the plasma treatment the wound healing was accelerated in diabetic rats compared to non-treated ones (F(2,15) = 11.33, H.R: 34%, P < 0.001). The plasma treatment accelerated the wound healing process drastically so that 7 days after plasma treatment the wound area of the rats reached its half value in comparison with the first day before plasma treatment (F(2, 15) = 11.33, H.R: 58%, p < 0.001). Moreover, 7 days after the plasma treatment, it reduced the wound area more than helium treatment (F(2, 15) = 6.35, H.R: 70% (compared to helium treatment), P < 0.01). 15 and 30 days after the plasma treatment the wound healed completely in plasma treated diabetic rats (F(2, 15) = 11.33, H.R: 95%, p < 0.001) showing a significant change in comparison to helium gas treatment (F(2, 15) = 11.33, H.R: 85% (compared to helium treatment), P < 0.001).

Figure 1(D) shows the wound contraction during various days of treatments.

### Plasma enhanced formation of epidermal layer, cell proliferation and neovascularization

**Figure 2:**
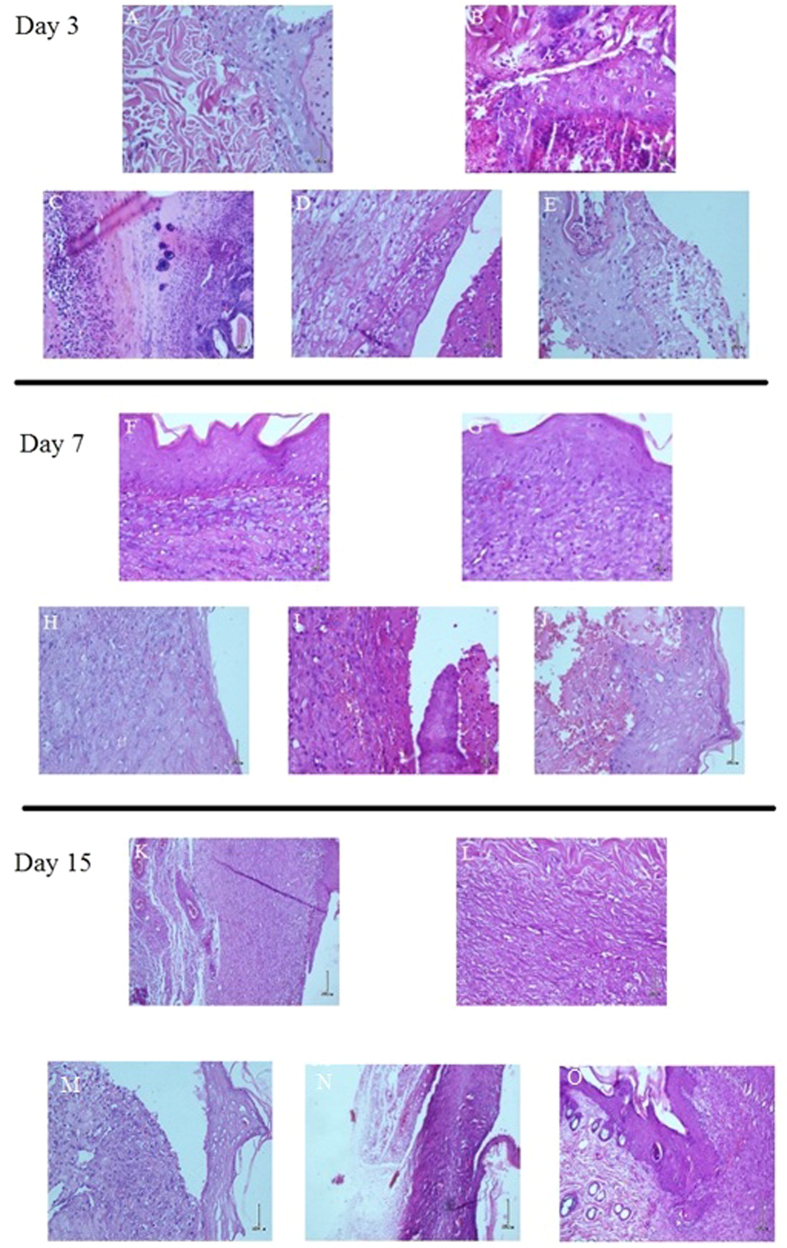
Histological aspect of the rat’s tissue. (**A–E**) Day 3, (**F–J**) day 7, (**K–O**) Day 15. The magnification of the images are 40X. For each day, Top row from left to right :non diabetic-control & non diabetic-plasma treatment. Bottom row from left to right: diabetic-control, diabetic-helium gas treatment & diabetic-plasma treatment.

Figure 2(A–E) shows the histological microscopic image from the wound at day 3 after helium gas and plasma treatment. The histological analysis showed some histomorphological differences between tissues taken from plasma-treated and control groups. The major diffrence was apparent at the epidermal layer. A new epidermal layer formed 3 days post-plasma treatment in diabetic animals (H.S: 2.3 ± 0.1, P < 0.05) which did not appear in control rats (H.S: 0.3 ± 0.1, P < 0.05). From the prospective of cellular study, the plasma induced fibroblast cell prolifration and thickness in cell walls in about 10 cell layers from skin surface (H.S: 2.7 ± 0, P < 0.05). Furthermore helium gas treatment caused the formation of a thin epidermal layer compared with non-treated rats (H.S: 0.7 ± 0.1, P < 0.05).

The histological image of the sampled tissues taken 7 days after the treatment has been shown in Fig. 2(F–J). The most important result that can be obtained from the image is the formation of epidermis after implantation of primary epidermal layer after plasma treatment (H.S: 3 ± 0, P < 0.01). In addition, it can be demonstrated that plasma treatment induced neovascularization largely in the dermis layer (H.S: 2.7 ± 0, P < 0.01). Moreover, plasma stimulated the production of collagen in the dermis layer (H.S: 2.3 ± 0.1, P < 0.05). Another interesting observation is the formation of a keratin layer in the skin surface which plays an important role in the wound healing process (H.S: 2.3 ± 0.1, P < 0.01). In addition, granulation tissue filled the incision space (H.S: 3 ± 0, P < 0.01). Furthermore, fibroblast cellular proliferation was reduced in this stage (H.S: 2 ± 0.1, P < 0.05 (compared to plasma treatment group 3 days after the plasma treatment)). The helium gas treatment improved cell proliferation (H.S: 1.3 ± 0.1, P < 0.05) and epidermis re-growth (H.S: 1.6 ± 0.6, P < 0.05), but less than plasma treatment (P < 0.05).

Figure 2(K–O) shows the histological image of the tissue taken from the rats 15 days after the treatment. Large full-fledged epidermis layer can be observed because of the plasma treatment in diabetic models (H.S: 2.3 ± 0.6, P < 0.05). Moreover, the formation of granulation tissue (H.S: 2.3 ± 0.3, P < 0.05) and keratin layer (H.S: 1.3 ± 0.2, P < 0.05) is clearly visible. The most important point obtained from the image is the new hair follicle which appeared in the epidermis and dermis layer allowing the hair growth in Fig. 1(D). Although the helium gas treatment did not heal the wound completely, it was effective in formation of the epidermis layer (H.S: 2.1 ± 0.1, P < 0.05).

### Plasma activates TGF-β1 cytokine

**Figure 3:**
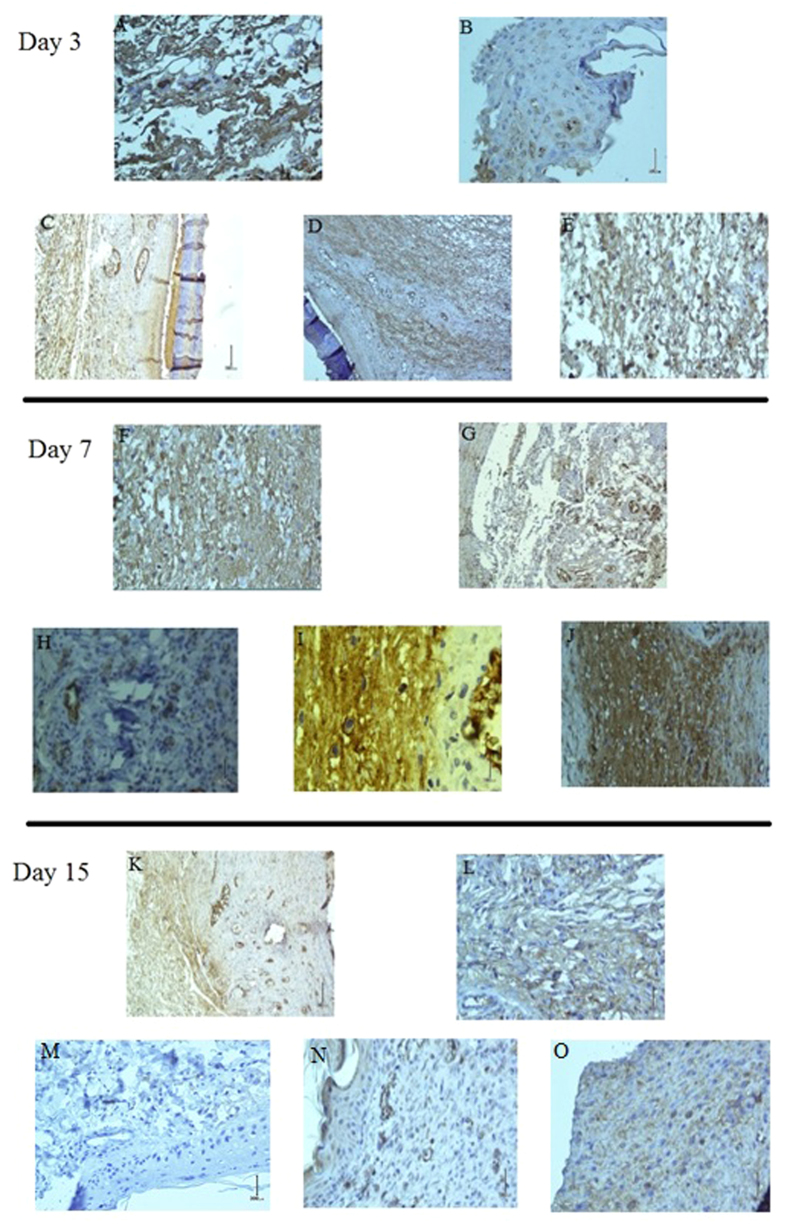
Tissue sections were immnunohistochemically stained with TGF-β1 antibody and showed with contrast against the background. (**A–E**) Day 3, (**F–J**) day 7, (**K–O**) Day 15. The magnification of the images are 40X. For each day, Top row from left to right :non diabetic-control & non diabetic-plasma treatment. Bottom row from left to right: diabetic-control, diabetic-helium gas treatment & diabetic-plasma treatment.

The immunohistochemistry analysis of the tissue samples is shown in Fig. 3. As shown in the figure there is no significant change in the amount of TGF-β1 released 3 days after the plasma treatment (IHC.S: 2 ± 0.6, P > 0.05). However, a major difference can be observed 7 days after the plasma treatment which is demonstrated in Fig. 3(F–J). These figures show that the blood plasma cells are the major cells that release cytokines in the diabetic wound tissue after the non-thermal plasma treatment (IHC.S: 3.3 ± 0.6, P < 0.01). Moreover, it can be observed that helium gas induced TGF-β1 release, but less than plasma treatment (IHC.S: 2.4 ± 0.3, P < 0.01). The immunohistochemistry image of the tissue taken 15 days after the plasma treatment is shown in Fig. 3(K–O). These figures show that the amount of cytokines was reduced significantly compared to 7 days after the plasma treatment (IHC.S: 1.7 ± 0.3, P < 0.01). Another interesting point obtained from the immunohistochemistry images is the aggregation of TGF-β1 on the endothelial cell walls after the plasma treatment.

### Active species produced in plasma medium

**Figure 4:**
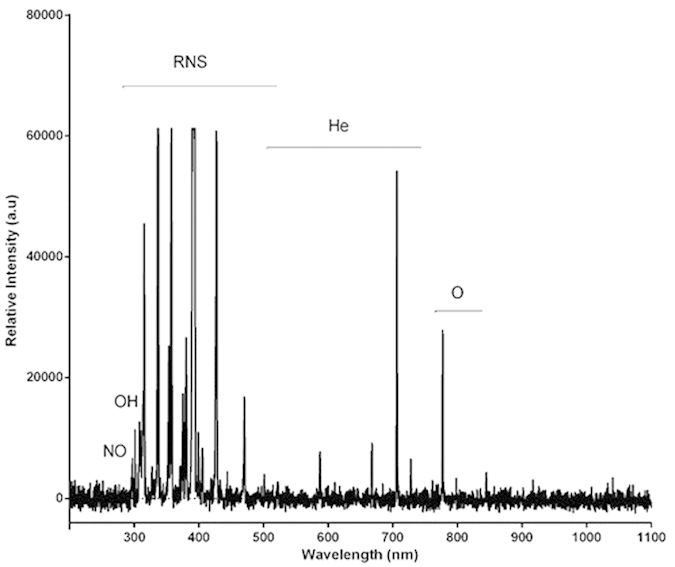
Optical Emission Spectroscopy collected from the plasma.

Figure 4 indicates the constituent particles in plasma, suggesting that a great number of ions and radicals were produced inside plasma. The major particles produced in plasma entail activated nitrogen species (ions, atoms) ranging from 300 to 500 nm. Another key species in plasma is oxygen created at 777 nm. Moreover, there are hydroxyl and nitric oxide molecules produced at 310 and 297 nm, respectively. These free radicals and chemical species play an important role in the interaction between plasma and cells.

## Discussion

To the best of our knowledge, the data obtained in this report reveal some new aspects among the few study designed to demonstrate the effect of non-thermal atmospheric pressure plasma on wound healing in metabolic disorders. Firstly, the results showed a low wound healing rate in diabetic animals compared to non-diabetic ones. Secondly, the results revealed that plasma treatment enhanced the wound healing rate in the non-diabetic model. Finally, the results showed that in diabetic animals the wound contraction occurred after the plasma treatment in contrast to untreated wounds. In addition, histological analysis showed the formation of the epidermis layer, neovascularization and cell proliferation. Moreover, the immunohistochemistry analysis indicated that the plasma treatment caused TGF-β1 cytokine release by cells in the tissue medium.

There are several factors which cause impairment in diabetic wound healing process. One of the main reasons for this issue is poor blood circulation in the tissue which leads to the lack of sufficient nutrients and oxygen in the wound closure area^18^. Therefore, neovascularization is a most essential event in diabetic wound healing^19^. Consistent with our study, the results confirmed that the plasma exposure induced neovascularization after 7 days of treatment. In confirmation of our findings, angiogenesis induced by plasma treatment has been reported by Hirata *et al.*^20^ and Fuxiang Ye *et al.*^21^ in burn wounds and chronic wounds respectively. While, these reports hypothesized that the reactive agents in plasma induced activation of growth factors accomplished by angiogenesis^20^,^21^, our outcomes revealed that the plasma may activate TGF-β1 growth factor which causes the release of this growth factor in the tissue medium. Lack of TGF-β1 as the outcome of cell dysfunction seen in diabetes mellitus is one of the major reasons in impairment of wound healing^22^. By analyzing the immunohistochemistry images of the rat’s tissue it can be obtained that 7 days after the plasma treatment the cells released the growth factor in the tissue medium. Moreover, the results revealed that most of the growth factor aggregated around the endothelial cells. Ferrari *et al.*^23^ stated that this may lead to enhanced angiogenesis which is in consistent with our findings.

In contrast to the normal wound closure events of keratinocyte cell migration that is seen after a few days, it was shown by Graves *et al.*^24^ that in diabetic patients, high glucose levels in the blood caused the over expression of the FOXO1 gene which leads to increase in the level of CCL20 protein instead of TGF-β1. This process frustrated the migration of keratinocyte cells in the wound closure area. The present study revealed that plasma treatment induced the generation of TGF-β1 which may lead to enhancement of keratinocyte cell migration and the formation of the keratin layer above the epidermis layer in diabetic rats.

Plasma generates reactive ions, molecules and free radicals such as atomic oxygen (O), hydroxyl (OH), nitrogen molecule (N_2_), super oxide anion (O_2_^−^) and nitric oxide (NO). It’s widely believed that these reactive agents play an important role in the interaction between the plasma and cells. Our results demonstrated the cellular inflammation which takes place predominantly in the epidermis layer and is induced by the interaction between the reactive species and the cells. Meturuki *et al.*^25^ suggested that NO activated the TGF-β1 cytokine by S-nitrosation of Latency-Associated Peptide. They also stated that NO activated the MAPK pathway which is involved in the cellular inflammation-proliferation process and plays a crucial role in wound healing. Moreover, reactive free radicals play a vital role in angiogenesis by activating the vascular endothelial growth factors^26^. In addition, reactive species participate in wound sterilization by the elimination of bacteria, fungi and viruses on the surface of the wound closure^27^.

In conclusion, this report demonstrates the effect of non-thermal atmospheric pressure plasma on diabetic wound healing. The results showed that the plasma therapy accelerated wound healing. Moreover, plasma treatment induced cell proliferation, epidermal layer formation and keratinocyte migration. In addition, immunohistochemistry analyses revealed that the cells released TGF-β1 cytokine which is vital for diabetic wound healing. The outcome of this study demonstrates a possible treatment effect of non-thermal atmospheric plasma for wound healing in diabetes mellitus in the future.

## Material and Method

### Ethics statement

All animal maintenance and procedures were in accordance with recommendations established by the Animal Ethics Committee of Tehran University of Medical Sciences as well as the United States NIH guidelines (publication no. 85–23). The protocols were approved by the Ethics Committee of Tehran University of Medical Sciences. All surgeries were performed under deep anesthesia, and all efforts were made to minimize suffering.

### Plasma setup

**Figure 5:**
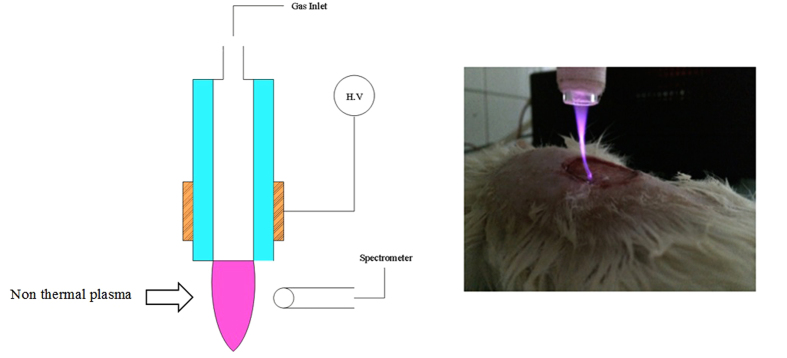
Schematic of plasma setup during an experiment (Left). Real discharge photo of the skin graft (Right).

The plasma jet consisted of a Pyrex tube (ID: 2 mm and OD: 4 mm) as an insulating tube nozzle. Copper wire as power electrode was wrapped around the glass tube with 10 mm width as nozzle. The distance between the nozzle tip and powered electrode was 10 mm. The power of electrode was driven by a pulsed DC high voltage connected to a high voltage power supply. The applied voltage, repetition frequency and duty ratio were 8 kV, 6 kHz and 15%, respectively. The feeding gases for this study were 99.999% pure helium (He) with 2 lit/min gas flow rate. A distance from the nozzle to wound surface was adjusted as 20 mm for a direct contact between plasma plumes and wound. The plasma was exposed for 10 minutes for each plasma treated group. The apparatus is illustrated in Fig. 5.

### Animal study

The effect of non-thermal atmospheric plasma was evaluated on 25 male wounded adult rats (250 ± 50 gr) in 5 groups. The rats were divided into the diabetic and non-diabetic model groups. The diabetic group consisted of 3 subgroups which were those treated by plasma, those treated by helium gas, and the third group placed as the control group which was not treated during any days of the experiment. The non-diabetic group consisted of 2 subgroups, one group exposed to plasma and the other group set as negative control without any treatment.

Diabetes was induced in the animals by intravascular injection of Streptozocin (Sigma Co., USA) with dosage of 60 mg/kg. The blood glucose of the diabetic group was measured after 3 days and the level of 360 ± 20 mg/dl for blood glucose was determined as the suitable level for diabetic mellitus in rats. After general anesthesia by injection of 80:10 mg/kg Ketamine:Xylazine (Alpha Co.) was given, a circular (2 mm in diameter) full-thickness skin wound at back (dorsum) of the rat were made. Moreover, the rats were caged individually in an air-conditioned room. All of the groups were observed daily from days 0 to 30 after wounding and the wound healing rates (H.R) were reported.

### Histological analysis

In addition to recording all detailed observations of the specimens, the formalin-fixed specimens were analyzed using microscopic histological techniques. The biopsied sections were processed for hematoxylin and eosin staining using the standard protocol^28^. Our pathologists were blinded to all group designations throughout the study. The histological image were scored based on the Abramov score method^29^. In this regard, Histological Score (H.S) was evaluated and reported in Table 2 according to six criterion: Acute inflammation (0:None, 1:Scant, 2:Moderate, 3:Abundant), Granulation tissue fibroblast maturation (0:Immature, 1:Mild maturation, 2:Moderate maturation, 3:Fully maturate), Collagen deposition (0:None, 1:Scant, 2:Moderate, 3:Abundant), Epidermis formation (0:None, 1:Partial, 2:Complete but immature or thin, 3:Compelete and mature), Neovascularization (0:None, 1:Up to five vessels per HPF, 2:6–10 vessels per HPF, 3:More than 10 vessels per HPF), and Keratin layer formation (0:None, 1:Partial, 2:Complete but immature or thin, 3:Compelete and mature).

### Immunohistochemistry assay

Wound tissue prepared on triplicate slides were used for immunohistochemical staining to analyze and demonstrate cytokine production using Transforming Growth Factor (TGF-β1) marker. Anti-TGF-β1 mouse monoclonal antibodies (abcam, Cambridge, MA) were employed. The slides were first warmed at 60 C to melt the paraffin wax and then hydrated through a graded ethanol series. The slides were heated up to 95 C in a modified citrate buffer to enhance antibody binding. The slides were then incubated with the primary antibody overnight at 4 °C. In order to remove excess primary antibody, the slides were washed the following day three times with phosphatebuffered saline (PBS) and then incubated with a rhodamine-conjugated anti-mouse secondary antibody for 2 h at room temperature and protected from light. To remove excess secondary antibody, three more washes with PBS were performed and then the slides were incubated for 3 min with a nuclear stain, Hoechst 33258 (0.05%). The slides were examined using a fluorescence microscope with attached Fluka Digital camera system. After the observation of the IHC stain slides, the images were analyzed by ImageJ software and IHC Profiler plugin and reported  in Table 3 30. The Immunohistochemistry was scored (IHC.S) automatically after the cell counting. The scored values are 1: Negative, 2: Low positive, 3: Positive and 4: High positive.

### Optical emission spectroscopy

Plasma spectroscopy was employed in order to find chemical species in the plasma. In this line, AvaSpec –ULS 2048 (Avantes Co) was used to collect the optical emission from the plasma. The spectrum of the irradiated light was collected in the interval 200–1100 nm with optical resolution of 0.5 nm. The spectra were subtracted from the dark baseline.

### Statistical analyses

The results were expressed as mean ± Standard Deviation (SD) of the wound area and calculated by GraphPad Prism Software. Student t-test was used for comparing the non-diabetes and diabetes controls also, non-diabetic control and non diabetic plasma treated groups. In addition, one way-ANOVA test was employed to compare the mean of each group with that of the control rats in diabetic plasma and helium treatment groups and scoring of the histological and immunohistochemistry staining. It should be noted that, P-values < 0.05 (*), P-values < 0.01 (**) and P-values < 0.001 (***) were considered as significant.

## Additional Information

**How to cite this article**: Fathollah, S. *et al.* Investigation on the effects of the atmospheric pressure plasma on wound healing in diabetic rats. *Sci. Rep.* **6**, 19144; doi: 10.1038/srep19144 (2016).

## Figures and Tables

**Table 1:** Blood glucose levels of the rats during the experiment days.

GroupName	Weight(gr)	Blood Glucose Level (mg/dl)			
		3 days beforethe treatment(Injection day)	Day 0–TreatmentDay	7 days after thetreatment	15 days after thetreatment
G1	237 ± 30	78.5 ± 6.2	82.7 ± 4.9	75.6 ± 4.7	87 ± 5.7
G2	228 ± 20	74.8 ± 7.4	78 ± 5.3	83.6 ± 5.6	72 ± 4.1
G3	204 ± 20	71.6 ± 3.1	395.5 ± 22.8***	405.2 ± 30.2***	395 ± 28.2***
G4	239 ± 25	83 ± 8.6	368.4 ± 24.6***	272 ± 25.6^**,#^	236.3 ± 23.2^**,##^
G5	214 ± 10	79.6 ± 9.5	361.6 ± 21.4^***^	383.4 ± 27.4^***^	219 ± 24.6^***,##^

G1: non-diabetic–control. G2: non- diabetic–plasma treatment. G3: diabetic- control. G4: diabetic–helium gas treatment. G5: diabetic- plasma treatment. *Compared with non-diabetic control (***p < 0.001, **p < 0.01), ^#^compared with diabetic control in each group (^##^p < 0.01, ^#^p < 0.05).

**Table 2:** Histological scoring of the tissue of the rats during the experiment days.

Day	Variable	G1	G2	G3	G4	G5	Significance
Day 3	Acute inflammation	1.3 ± 0.6	2.3 ± 0.6	0.7 ± 0.1	1.4 ± 0.6	2.7 ± 0.3	P < 0.05
	Granulation tissue fibroblast maturation	1.3 ± 0.3	1.3 ± 0.3	0.3 ± 0.1	1 ± 0	2.0 ± 0	P > 0.05
	Collagen deposition	1.3 ± 0.3	1.3 ± 0.1	0	1.6 ± 0.1	2.3 ± 0.6	P < 0.05
	Epidermis formation	1.3 ± 0	1.3 ± 0.6	0.3 ± 0.1	0.7 ± 0.1	2.3 ± 0.1	P < 0.05
	Neovascularization	0.7 ± 0.1	1.7 ± 0.6	0.3 ± 0.1	1.7 ± 0.3	2.0 ± 0.1	P > 0.05
	Keratin layer formation	1.7 ± 0.6	1.6 ± 0.6	0	0.7 ± 0.1	1.3 ± 0.2	P > 0.05
Day 7	Acute inflammation	2 ± 0.1	2 ± 0	1.3 ± 0.3	1.3 ± 0.1	2 ± 0.1	P > 0.05
	Granulation tissue fibroblast maturation	2.3 ± 0.6	2.6 ± 0.5	0.3 ± 0.1	1 ± 0.1	3 ± 0	P < 0.01
	Collagen deposition	0.7 ± 0.1	2.3 ± 0.5	1 ± 0.1	1.3 ± 0.1	2.3 ± 0.3	P < 0.05
	Epidermis formation	2.3 ± 0.6	2.3 ± 0.1	0.3 ± 0.1	1.6 ± 0.6	3 ± 0	P < 0.01
	Neovascularization	1 ± 0.1	2 ± 0	0.3 ± 0.1	1.6 ± 0.3	2.7 ± 0	P < 0.01
	Keratin layer formation	2.3 ± 0.3	2.3 ± 0.1	0	0.7 ± 0.1	2.3 ± 0.1	P < 0.01
Day 15	Acute inflammation	0.7 ± 0.1	0.6 ± 0.1	0.7 ± 0.1	1.3 ± 0.1	0.7 ± 0.1	P > 0.05
	Granulation tissue fibroblast maturation	1.7 ± 0.3	2.3 ± 0.3	0.3 ± 0.1	1 ± 0.3	2.3 ± 0.3	P < 0.05
	Collagen deposition	1.7 ± 0.3	2.3 ± 0.1	0.3 ± 0.1	1.7 ± 0.6	2.7 ± 0.3	P < 0.05
	Epidermis formation	2.3 ± 0.1	2.3 ± 0.6	0	2.1 ± 0.1	2.3 ± 0.6	P < 0.05
	Neovascularization	1 ± 0.1	0.7 ± 0.1	0.3 ± 0.1	1.7 ± 0.3	1.7 ± 0.1	P < 0.05
	Keratin layer formation	1.7 ± 0.6	1.6 ± 0.6	0	0.7 ± 0.1	1.3 ± 0.2	P > 0.05

G1: non-diabetic–control. G2: non-diabetic–plasma treatment. G3: diabetic- control. G4: diabetic – helium gas treatment. G5: diabetic- plasma treatment.

**Table 3:** The immunohistochemistry scoring of the tissues taken during the experiment days.

Treatment Day’s	G1	G2	G3	G4	G5	Significance
3 Day’s after the treatment	1.4 ± 0.3	1.7 ± 0.3	1 ± 0.1	1.7 ± 0.3	2.0 ± 0.6	P > 0.05
7 Day’s after the treatment	1.7 ± 0.6	2 ± 0.3	1 ± 0.1	2.4 ± 0.3	3.3 ± 0.6	P < 0.01
15 Day’s after the treatment	1.4 ± 0.3	1.7 ± 0.3	1 ± 0	1.7 ± 0.6	1.7 ± 0.3	P > 0.05

G1: non- diabetic – control. G2: non-diabetic – plasma treatment. G3: diabetic- control. G4: diabetic – helium gas treatment. G5: diabetic- plasma treatment.
